# Diet in Scleroderma: Is There a Need for Intervention?

**DOI:** 10.3390/diagnostics11112118

**Published:** 2021-11-15

**Authors:** Alexandra Maria Burlui, Anca Cardoneanu, Luana Andreea Macovei, Ciprian Rezus, Lucian Vasile Boiculese, Mariana Graur, Elena Rezus

**Affiliations:** 1 Department of Rheumatology and Rehabilitation, “Grigore T. Popa” University of Medicine and Pharmacy, 700115 Iasi, Romania; anca.cardoneanu@umfiasi.ro (A.C.); luana.macovei@umfiasi.ro (L.A.M.); elena.rezus@umfiasi.ro (E.R.); 2 Department of Internal Medicine, “Grigore T. Popa” University of Medicine and Pharmacy, 700115 Iasi, Romania; 3 Department of Preventive Medicine and Interdisciplinarity, “Grigore T. Popa” University of Medicine and Pharmacy, 700115 Iasi, Romania; vasile.boiculese@umfiasi.ro; 4 Department of Diabetes, Nutrition and Metabolic Diseases, “Grigore T. Popa” University of Medicine and Pharmacy, 700115 Iasi, Romania; mariana.graur@umfiasi.ro

**Keywords:** systemic sclerosis, diet, nutrition, relative fat mass, sodium, weight loss, malnutrition, digestive symptoms, pulmonary hypertension, disease activity

## Abstract

Systemic sclerosis (SSc) patients exhibit a plethora of risk factors for nutritional decline, including the presence of chronic inflammation and the progressive nature of disease-related multisystem involvement. The prevalence and consequences of nutritional decline in scleroderma are frequently underestimated, its management currently remaining a subject of debate. The main objective of the present study was to perform a detailed assessment of scleroderma patients’ diet as well as their eating habits and to describe the relationships with weight loss and malnutrition risk in the absence of professional nutritional counseling. Methods: We used a translated and validated version of the EPIC-Norfolk FFQ (European Prospective Investigation into Cancer and Nutrition Norfolk Food Frequency Questionnaire) to evaluate the patients’ diet and MUST (Malnutrition Universal Screening Tool) to investigate the risk of malnutrition. Disease activity was estimated using the EUSTAR-AI (European Scleroderma Trials and Research group Activity Index). Results: We included 69 patients with SSc, of which 42 underwent a detailed dietary assessment. Dietary factors were connected to body composition and digestive symptoms. We found high sodium intake and frequent suboptimal energy consumption in our study group, including patients with cardiopulmonary involvement. Liver transaminases were inversely correlated with the consumption of nuts and seeds. Malnutrition and weight loss were significantly associated with pulmonary hypertension, heart failure, albumin levels, and the extent of skin fibrosis, but not advanced age. Although the patients with EUSTAR-AI ≥ 2.5 were more frequently included in the moderate and high malnutrition risk categories, these results did not reach statistical significance. Conclusions: Currently, there is an unmet need for longitudinal and interventional research focusing on the long-term significance, ramifications, and management of nutritional impairment in SSc patients with various clinical manifestations. Our results indicate that scleroderma patients could benefit from personalized nutritional counseling in an interdisciplinary setting.

## 1. Introduction

Systemic sclerosis (SSc) is a rare chronic autoimmune disease mainly characterized by microvasculopathy, together with a widespread fibrosis of the skin and viscera [1]. SSc patients demonstrate an abundance of risk factors for nutritional decline, including the presence of chronic inflammation and the progressive nature of disease-related multisystem involvement [2,3,4].

Notably, it has been shown that gastrointestinal manifestations (occurring in up to 90% of patients and involving the entire digestive tract) are the most prevalent type of organ involvement after skin fibrosis [5]. Patients’ diet has been studied mainly with respect to its relationship with the appearance or aggravation of disease-related digestive symptoms [6]. Moreover, certain dietary recommendations have been proposed for the alleviation of such digestive manifestations as gastroesophageal reflux, dysphagia, delayed gastric emptying or gastroparesis, and bowel involvement (leading to constipation or diarrhea), yet there is a lack of interventional studies specifically proving their efficacy in SSc [5].

Malnutrition has been linked to a more aggressive course of the disease and could derive from complications related to gastrointestinal involvement, as well as from numerous extra-digestive factors [6]. Data pertaining to weight loss, malnutrition risk, and nutritional management in scleroderma remain scarce [6]. Nevertheless, unintentional weight loss was included in the Medsger scoring system as an indicator of severity within the general symptoms section, suggesting the need for careful monitoring and correlation with other beacons of disease progression [7]. In a Mexican study of 220 patients with SSc followed over a 10-year period, mortality risk was associated with the Medsger score for general symptoms and severe malnutrition [8].

The main objective of the present study was to perform a comprehensive assessment of scleroderma patients’ diet as well as their eating habits and to describe the relationships with weight loss, biochemical parameters, and malnutrition risk in the absence of previous professional nutritional counseling.

## 2. Materials and Methods

We conducted a prospective observational study in which we included adult individuals who fulfilled the American College of Rheumatology/European League Against Rheumatism (ACR/EULAR) 2013 classification criteria for SSc [9] and were admitted to the rheumatology department for reevaluation. We excluded the patients who were pregnant; those who had benefited from professional nutritional counseling and were following a specific diet (in order to either lose or gain weight); those who had neoplasia (confirmed or suspected), overlap syndromes, or mixed connective tissue disease; those with incomplete data; as well as those who refused to participate.

Such characteristics as the disease phenotype according to LeRoy (limited cutaneous SSc-*lcSSc* and diffuse cutaneous SSc-*dcSSc*), the modified Rodnan skin score (mRSS), and organ involvement were taken from patients’ charts. The risk of malnutrition was estimated according to MUST scores (Malnutrition Universal Screening Tool). The patients were classified as having a low risk (MUST score = 0), a moderate risk (MUST score = 1), or a high risk of malnutrition (MUST score ≥ 2, needing prompt nutritional intervention).

Disease activity was estimated using the EUSTAR-AI (European Scleroderma Trials and Research group Activity Index) [10,11].

We performed the dietary analysis using a translated, adapted, and validated variant of the European Prospective Investigation into Cancer and Nutrition Norfolk Food Frequency Questionnaire (EPIC-Norfolk FFQ) [12]. The questionnaire included a first part containing 130 items (pertaining to the rate of consumption of certain foods, and alcoholic and non-alcoholic beverages), and a second part regarding the patients’ choice of breakfast cereal, the type and amount of milk consumed, food preparation (grilling, frying, roasting, baking), as well as the fat used in cooking the meals and the consumption of visible fat on the meat. Lastly, the questionnaire recorded the intake of dietary supplements.

The information derived from the EPIC-Norfolk FFQ was included in a Microsoft Excel database and subsequently processed using the FETA software version 2.53 for Windows. This resulted in an estimation of the average daily energy intake, macronutrients, and micronutrients together with the dietary consumption by food groups [13].

The estimated energy requirement (EER) was calculated as follows [14]:

- Women: EER (kcal/day) = 2403 − 7 × (age − 19);
- Men: EER (kcal/day) = 3067 −10 × (age − 19).

Regarding sodium consumption, we compared the results obtained within the study group to the adequate intake values (AI) and the tolerable upper intake levels (TUI) according to the patients’ age. The amounts of dietary fiber and vitamin D consumed were compared to the AI values according to age and gender [14].

An additional questionnaire was used to assess the patients’ eating habits (the number of daily meals, the most substantial meal, as well as regular nighttime meals or snacks), the presence of digestive symptoms, and the average number of hours of sleep for each patient. We recorded the subjects’ unintentional weight loss over the previous 6 months. The participants’ body mass and BMI noted at the moment of diagnosis (W_dg_, BMI_dg_) were taken from the hospital archives and previous databases.

The anthropometric parameters evaluated were height (H), weight (W), abdominal circumference (AC), hip circumference (HC), body mass index (BMI, calculated as W/H^2^), mid-arm circumference (MAC), the waist/hip ratio (WHR), and the relative fat mass (RFM). The latter was calculated as follows [15]:

- Women: RFM_f_ = 76 − (20 × H/AC);
- Men: RFM_m_ = 64 − (20 × H/AC);
  or
- RFM = 64 − (20 × H/AC) + (12 × Gender) (Female = 1, Male = 0).

The BMI classes were defined according to the WHO (World Health Organization) as underweight for a BMI under 18.5 kg/m^2^, eutrophic for values between 18.5 and 24.9 kg/m^2^, pre-obesity for a BMI between 25.0 and 29.9 kg/m^2^, and obesity in the case of values ≥ 30 kg/m^2^ [16].

Blood samples (5 mL venous blood/patient) were drawn to evaluate the following biochemical parameters: serum albumin, total protein, C-reactive protein (CRP), total cholesterol, triglycerides, creatinine, vitamin D, aspartate aminotransferase (AST), and alanine transaminase (ALT). The blood samples were analyzed within the hospital laboratory. Vitamin D insufficiency was defined as serum levels of 10–30 ng/mL, whereas deficiency was determined in patients with titers lower than 10 ng/mL.

The statistical analysis of the data was performed using IBM SPSS Statistics version 23 for Windows. The statistical significance threshold was set at *p* ≤ 0.05.

All patients signed an informed consent form in order to participate. The present study was conducted according to the guidelines of the Declaration of Helsinki and approved by the Ethics Committees of the “Grigore T. Popa” University of Medicine and Pharmacy (1/4.04.2017) and the Clinical Rehabilitation Hospital of Iasi, Romania (1/2.05.2017).

## 3. Results

### 3.1. General Characteristics

**Figure 1:**
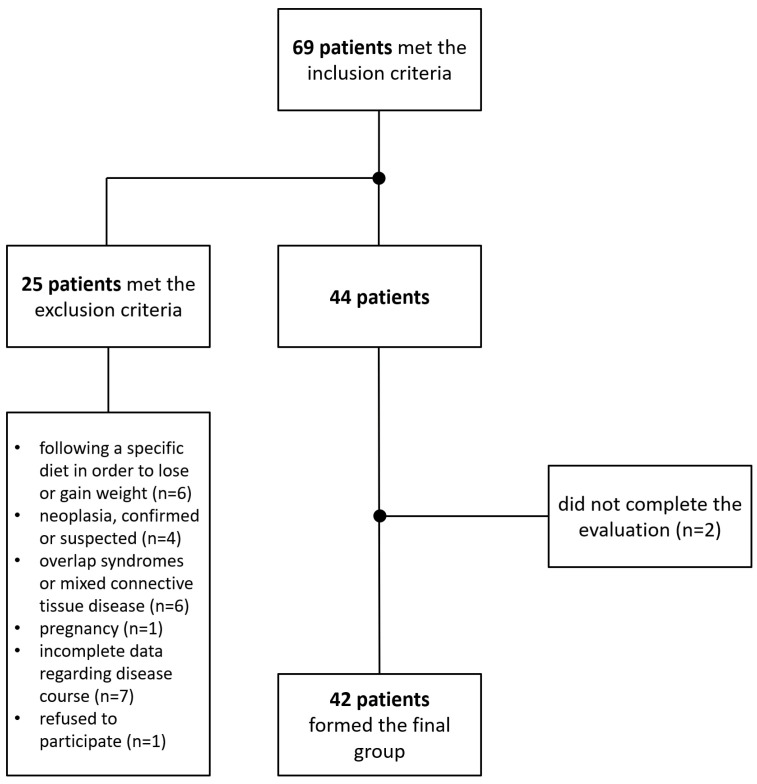
Flow diagram of the inclusion and exclusion processes leading up to the final study group.

A total of 69 adult Caucasian patients with SSc met the inclusion criteria. After eliminating the subjects who met the exclusion criteria for the present study or did not complete the evaluation, 42 patients formed the final study group (Figure 1) and underwent a comprehensive dietary assessment.

According to the LeRoy classification, 24 patients had lcSSc, whereas 18 were diagnosed with dcSSc. Women constituted the majority of the study cohort (F:M ratio = 6:1) (Table 1). We noted mRSS values ≥ 20 points in nine patients (21.4%), this finding being more prevalent in patients with dcSSc (*p* < 0.001). Disease duration did not differ according to disease phenotype.

The most prevalent SSc-related cardiopulmonary manifestation was ILD (interstitial lung disease). ILD was significantly more frequent in dcSSc (*p* = 0.001), although without statistical significance, PAH was more prevalent in lcSSc and CHF (chronic heart failure) in dcSSc.

Patients with dcSSc were more likely to report the presence of digestive symptoms (*p* = 0.007). The most prevalent digestive manifestation was gastroesophageal reflux disease (GERD, 73.8%). Although dysphagia was significantly more prevalent among the dcSSc subgroup (*p* = 0.005), we did not find a significant relationship between other digestive symptoms and disease phenotype. We did not identify a higher frequency of dysphagia in the elderly (participants aged over 65 years).

The subjects who reported the presence of dysphagia and early satiety had higher mRSS values (*p* < 0.001 and *p* = 0.003). However, the abovementioned digestive symptoms were not found to be significantly more prevalent in dcSSc.

The participants with EUSTAR-AI values over 2.5 were significantly more likely to also exhibit digestive symptoms (*p* = 0.050). Moreover, the dcSSc subgroup demonstrated significantly higher EUSTAR-AI values compared to lcSSc (*p* = 0.001).

The mean values for all anthropometric parameters were lower in patients with mRSS ≥ 20, yet only BMI and body mass demonstrated significant differences in this respect (*p* = 0.005 and *p* = 0.042). Underweight was also found to be more prevalent in patients with mRSS ≥ 20 (*p* = 0.048).

The subjects who reported sleeping less than 7 h/day on a regular basis had higher AC values (*p* = 0.034).

Early satiety and decreased appetite were more frequently noted in subjects who were underweight (*p* = 0.002 and *p* < 0.001). Subjects with early satiety demonstrated a significantly lower body mass (*p* = 0.011).

EUSTAR-AI values were not significantly correlated with the anthropometric parameters evaluated in our study group. Moreover, there were no notable differences with respect to anthropometric data between patients with EUSTAR-AI scores over or under 2.5 points.

The patients’ body mass recorded at the moment of diagnosis (W_dg_) was higher than the value measured at the time of the study. The prevalence of excess weight at diagnosis (BMI_dg_ ≥ 25 kg/m^2^) demonstrated a markedly higher prevalence compared to the present assessment (*p* < 0.001). The distribution according to BMI_dg_ revealed a higher frequency of obesity (albeit without statistical significance), as well as the absence of an underweight category.

### 3.2. Unintentional Weight Loss and Malnutrition Risk

Unintentional weight loss was found in 17 patients (40.5%). The amount of weight loss identified in our study group varied between 0 and 24 kg (2.95 ± 5.22 kg). The dcSSc subgroup exhibited higher median values for unintentional weight loss compared to lcSSc. However, these results did not reach statistical significance. Nevertheless, mRSS was correlated with weight loss (*r* = 0.306, *p* = 0.049), with patients with mRSS ≥ 20 exhibiting a significantly higher prevalence of unintentional weight loss (*p* = 0.019). Disease activity (EUSTAR-AI) was not significantly linked to weight loss.

We did not find notable relationships between weight loss and dysphagia (in terms of frequency or the amount of weight lost). However, subjects with early satiety demonstrated an increased risk of weight loss (RR = 5.550; 95% IC: 4.723–7.881). Additionally, this subgroup had a higher prevalence of weight loss of more than 10% of their initial body mass (*p* = 0.026).

In lcSSc patients, PAH was associated with weight loss of more than 10% of the patients’ initial body mass (*p* = 0.013). CHF was linked to weight loss of more than 10% in subjects with dcSSc (60% versus 7.7%; chi^2^, *p* = 0.017).

We did not identify a significant relationship between EUSTAR-AI and unintentional weight loss, including when comparing patients with values over 2.5 points to the rest of the group.

According to MUST, six participants (14.29%) had a high risk of malnutrition and five (11.9%) were in the moderate-risk category, whereas the majority (31 patients, 73.81%) showed a low risk of malnutrition. The low-risk category demonstrated a preponderance of the lcSSc phenotype, yet without statistical significance.

The patients classified as low risk according to MUST demonstrated significantly lower mRSS values compared to the rest of the group (*p* = 0.020). Although patients with EUSTAR-AI ≥ 2.5 were more likely to have a MUST score ≥ 1 (38.9% versus 16.7%), these results did not reach statistical significance.

MAC values were significantly lower in patients with MUST scores ≥ 1 (corresponding to the medium and high malnutrition risk categories) compared to the rest of the study group (*p* = 0.019). We did not find a significant connection between old age (over 65 years) and a higher malnutrition risk in our patients. This was also true for disease duration.

The lack of appetite and early satiety were significantly linked to high malnutrition risk (*p* < 0.001 and *p* = 0.002). Additionally, patients with dysphagia were more likely to be included in the moderate- or high-risk MUST categories (*p* = 0.023).

The presence of PAH was associated with higher MUST scores in lcSSc (*p* = 0.050) as well as dcSSc (*p* = 0.026). Additionally, patients with dcSSc and CHF were more likely to exhibit MUST scores ≥ 1 (*p* = 0.026).

### 3.3. Biochemical Parameters

The values of albumin, total protein, vitamin D, CRP, AST and ALT, total cholesterol, triglycerides, and blood glucose are shown in Table 2.

The participants who were underweight (BMI < 18.5 kg/m^2^) were more likely to exhibit hypoalbuminemia (*p* < 0.001). Additionally, albumin values were significantly correlated with the patients’ body mass (*r* = 0.396, *p* = 0.009), BMI (*r* = 0.432, *p* = 0.004), RFM (*r* = 0.315, *p* = 0.042), and MAC (*r* = 0.322, *p* = 0.028), but not AC, WHR, or mRSS. Unintentional weight loss and MUST scores were inversely correlated with serum albumin (*p* = 0.031 and *p* = 0.001, respectively). Patients with high malnutrition risk had the lowest albumin titers of the three MUST categories (*p* = 0.012). The EUSTAR-AI values were not significantly linked to albumin titers in our study group.

Vitamin D levels were under 30 ng/mL in most patients (40 subjects, 95.2%), of which 27 participants had values under 10 ng/mL (64.3%). Although titers were lower in the dcSSc subgroup compared to the lcSSc group, these results did not reach statistical significance. However, vitamin D was correlated with mRSS (*r* = −0.338, *p* = 0.028). Serum vitamin D levels were lower in the subgroup with a EUSTAR AI of under 2.5, albeit without statistical significance.

Serum CRP was significantly correlated to the patients’ WHR (*r* = 0.319, *p* = 0.004). We found significant links between serum triglycerides and AC, as well as the patients’ WHR (*p* = 0.009 and *p* = 0.004). The correlation between total cholesterol levels and BMI approached statistical significance (*p* = 0.060).

We did not find a notable association between CRP or serum lipids and the participants’ RFM. The latter was significantly correlated with AST (R = 0.327, *p* = 0.034) and ALT values (*r* = 0.347, *p* = 0.024).

Creatinine levels were significantly associated with age (*r* = 0.517, *p* < 0.001), mRSS (*r* = 0.488, *p* = 0.001), and EUSTAR-AI (*r* = 0.320, *p* = 0.039).

### 3.4. Eating Habits and EPIC-Norfolk FFQ

Overall, patients with digestive symptoms reported less frequent nighttime snacks (11.8% versus 37.5%), albeit without statistical significance. Nonetheless, patients with GERD were significantly less likely to consume nighttime meals or snacks (*p* = 0.041).

All participants belonging to the subgroup with early satiety reported consuming less than three meals/day. Moreover, patients with early satiety were more likely to have only one meal/day (40% compared to 21.6% in the rest of the group). Similarly, subjects with decreased appetite were more likely to consume only one meal/day (50% versus 21.1%). The number of daily meals was not significantly correlated with MUST scores or the amount of weight lost in our study group.

All 42 patients in the detailed assessment group completed the dietary evaluation by the EPIC-Norfolk FFQ.

#### 3.4.1. Foods and Beverages

We found that the participants who had suffered unintentional weight loss consumed significantly lower amounts of sugars, preserves, and snacks. However, this subgroup reported a higher consumption of soups (Table 3).

The patients with GERD reported consuming more fats and oils compared to the rest of the group (*p* = 0.045). The subjects with dysphagia ate larger amounts of sweets, preserves, and snacks (*p* = 0.014).

The MUST low-risk subgroup reported consuming higher amounts of fruits and vegetables (*p* = 0.041 and *p* = 0.025), yet we did not find notable relationships with other foods or beverages.

The consumption of fats and oils was connected to higher blood triglycerides (*r* = 0.440, *p* = 0.004) and total cholesterol (*r* = 0.315, *p* = 0.042).

AST and ALT were inversely correlated with the intake of nuts and seeds (*r* = −0.315, *p* = 0.022, and *r* = −0.423, *p* = 0.004, respectively). We did not obtain significant relationships between food intake and creatinine level.

The daily consumption of alcohol varied between 0 and 9.55 g/day. We found that male subjects consumed significantly more alcohol than females (*p* = 0.031). This was also true for patients with excess abdominal fat according to AC and WHR (*p* = 0.041 and *p* = 0.036). Moreover, we found a significant correlation between the consumption of alcoholic beverages and RFM values (*p* < 0.001).

The patients who reported sleeping less than 7 h/day consumed significantly more fats and oils than the rest of the group (*p* = 0.034). Although these subjects also had higher intakes of sugars, preserves, and snacks, these results did not reach statistical significance.

#### 3.4.2. Energy and Macronutrients

According to the data derived from the EPIC-Norfolk questionnaire, the mean value of the average daily energy intake was 1944.05 kcal. Overall, the daily caloric intake as processed by FETA was lower in the study group than the mean EER (*p* = 0.024). Compared to EER values, caloric intakes were lower in 22.8% of the participants with PAH, 72.2% of the ILD subgroup, and in 57.1% of participants with CHF. Subjects with PAH were more likely to demonstrate lower caloric intakes than the EER (*p* = 0.042).

Urban dwellers consumed more carbohydrates (*p* = 0.039) and fats (*p* = 0.029) than patients residing in rural areas. Albeit without statistical significance, mean caloric intakes were higher in patients who had experienced unintentional weight loss (Table 4).

According to BMI, patients who were either underweight or eutrophic were significantly more likely to report a daily caloric intake lower than the EER (*p* = 0.021). The subjects with a BMI ≥ 25 kg/m^2^ (pre-obese and obese) consumed less amounts of protein (*p* = 0.046) and reported a higher intake of sucrose and total sugars (*p* = 0.031 and *p* = 0.041).

The patients who reported having decreased appetite were significantly more likely to have low caloric intakes (less than the EER; *p* = 0.013). The subjects with dysphagia exhibited a significantly higher mean caloric intake than the rest of the study cohort (*p* = 0.036).

**Figure 2:**
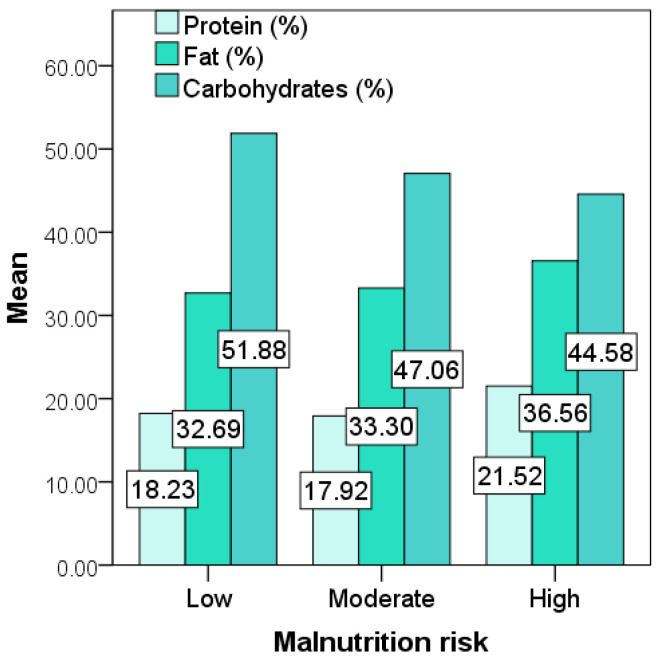
Macronutrient energy contributions with respect to malnutrition risk (MUST).

The participants with a low malnutrition risk reported a higher carbohydrate intake (expressed as % of daily caloric intake) than the rest of the cohort (*p* = 0.039). In the absence of statistical significance, the high-risk subgroup exhibited the highest mean macronutrient energy contribution for dietary fat. The intake of protein, lipids, and carbohydrates (expressed as % of the daily caloric intakes) with respect to the risk of malnutrition is illustrated in Figure 2.

The subjects who reported sleeping less than 7 h/day on a regular basis consumed significantly larger amounts of saturated fats (*p* = 0.040), as well as total fat (expressed as % of the daily caloric intake; *p* = 0.024) and sugars (*p* = 0.022).

Total serum protein levels were significantly associated with the mean intakes of dietary proteins (*r* = 0.330, *p* = 0.033).

#### 3.4.3. Micronutrients and Dietary Fiber

The average daily values for micronutrient consumption (from dietary sources and supplements) in the study cohort are shown in Table 5. The participants who had suffered unintentional weight loss reported significantly higher intakes of sodium and chloride. Moreover, the daily consumption of sodium was higher than the AI values in 41 patients (97.6% of the group) and superior to the TUI levels in 27 cases (64.3% of the group).

All the patients with PAH and CHF exceeded the AI values for sodium. Sodium consumption higher than the TUI was found in 57.1% of subjects with CHF and 88.9% of the participants with PAH.

The dietary intake of vitamin D was significantly lower than the AI values (according to the patients’ age and gender, *p* < 0.001). Only two patients in our study group (4.76%) consumed the adequate daily amount of dietary vitamin D, yet did not demonstrate normal serum vitamin D levels. Almost half of the group had been taking vitamin D supplements in various amounts (20 patients, 47.62%), of which 13 were taking supplements on a daily basis (30.95%) and nine (21.43%) were under 1 μg alfacalcidol/day (the maximum dose found in our study group). Serum vitamin D titers were not significantly connected to intakes from diet or supplements (including the prevalence of deficiency).

The average intake of dietary fiber varied between 3.63 and 46.61 g/day (18.05 ± 8.97 g/day). A subgroup of eight patients (19%) demonstrated a consumption of dietary fiber superior to the AI values with respect to their gender and age. However, we did not identify a significant relationship between the amount of fiber consumed and digestive symptoms (overall or for each recorded symptom).

## 4. Discussions

The prevalence and consequences of nutritional decline in scleroderma are frequently underestimated [17] and thus remain a subject of debate [18,19,20,21]. Largely due to the lack of scientific evidence on the matter, professional nutritional counseling is seldom regarded as a primary measure for the management of SSc-related weight loss, malnutrition risk, and gastrointestinal symptoms [22,23].

The EPIC-Norfolk FFQ has been used for detailed dietary assessment in different chronic conditions such as diabetes, cardiovascular diseases, and neoplasia, as well as in autoimmune diseases, the resulting software-processed data being linked to a variety of clinical manifestations and other parameters of interest [24,25,26,27,28]. We obtained several statistically significant relationships that have also been demonstrated in the general population regarding patients’ diet: alcohol consumption and male gender together with anthropometric indicators of excess abdominal adiposity [29,30,31], the connection between high BMI and an increased daily intake of sucrose and total sugars [32], as well as the relationship between sleep and food choices (which may be bidirectional) [33]. Another finding that was reported previously is the frequent consumption of excess sodium compared to the recommended intake [34]. Furthermore, decreased appetite was associated with a lower number of daily meals, similar to other studies [35].

Nevertheless, our SSc cohort exhibited certain differences from the diet and malnutrition-related patterns described in the general population. This includes the finding that subjects who had experienced weight loss consumed significantly larger amounts of sodium and chloride compared to the rest of the group, excess salt intake being commonly associated with weight gain [36]. The investigation of salt resistance in scleroderma patients could be useful in explaining this phenomenon, yet may not be advised for ethical reasons. In this respect, the evaluation includes the administration of diuretics, which could precipitate the appearance of scleroderma renal crisis [37].

Whereas it is often recommended that patients with chronic cardiovascular conditions follow a sodium-restricted diet, the latter often involves a poorer taste of the foods consumed, which explains the low adherence rates to a great extent [38,39,40,41]. In our study cohort, both CHF as well PAH participants consumed large amounts of sodium daily. The benefits of a low-sodium diet in cardiovascular diseases remain a subject of controversy [42,43]. In SSc, the potential advantages of dietary sodium restriction in the context of PAH and CHF are yet to be investigated by longitudinal studies.

Türk et al. evaluated the factors linked to higher MUST scores, obtaining a statistically significant relationship between the risk of malnutrition and ILD and gastrointestinal manifestations, but not PAH or overall cardiac involvement [19]. In our study cohort, the presence of PAH was connected to weight loss in lcSSc patients and malnutrition risk in both lcSSc and dcSSc. However, our results revealed an additional risk factor for nutritional impairment in SSc-related PAH, with those patients demonstrating a lower calorie consumption relative to the EER values. Although weight loss in obese patients with PAH has been proven to ameliorate certain cardiovascular risk factors in the general population, its effects on SSc-related PAH remain uncertain [44,45].

Malnutrition is frequent in CHF and has been shown to influence the patients’ vital prognosis [46,47,48]. With respect to CHF, which was more prevalent in dcSSc, we obtained statistically significant relationships with weight loss and malnutrition risk only in the abovementioned subgroup. However, patients with CHF were shown to consume less than the EER in over 50% of cases, indicating that diet may be a confounding factor in the relationship between CHF and nutritional decline in SSc.

A systematic review suggested a “15 percent rule” for scleroderma-related serious complications, finding a prevalence close to 15% for heart involvement, PAH, impaired lung function, arthritis, and myositis. Although malnutrition was considered for investigation as a severe manifestation of SSc, the authors did not describe the pertinency of the “15 percent rule” regarding this aspect in their results [49]. Interestingly, our study group exhibited a high malnutrition risk in a proportion nearing 15%. However, further research is needed to verify the abovementioned hypothesis.

EUSTAR-AI is a valuable tool in the assessment of disease activity in scleroderma and has been proven to be a good predictor of severity accrual [10,11]. We found statistically significant relationships between EUSTAR-AI and the diffuse phenotype (similar to previously published research), as well as concomitant digestive symptoms. Patients with a EUSTAR-AI ≥ 2.5 were more likely to be at risk of malnutrition and had lower vitamin D titers in our study group, albeit without statistical significance. EUSTAR-AI was not notably linked to body composition, weight loss or albumin levels. Nevertheless, EUSTAR-AI and nutritional parameters may adopt different variation patterns over time, requiring longitudinal studies to examine the dynamics of this relationship.

The presence of extensive skin involvement (mRSS over 20 points regardless of disease phenotype) was connected to lower body mass and BMI, with underweight being more prevalent in this subgroup. Notably, underweight was not significantly more frequent in patients with dcSSc in our study population. High mRSS values were also connected to unintentional weight loss and MUST scores. Similarly, Bagnato et al. [21] and Türk et al. [19] obtained significant relationships between the extent of skin involvement and malnutrition risk in their SSc cohorts.

Serum albumin is a reliable indicator of nutritional decline and was proven to correlate with malnutrition risk and unintentional weight loss in SSc and other chronic conditions, with our results mirroring those obtained in previous studies on the matter [18,50]. However, not all participants with underweight or a high malnutrition risk had hypoalbuminemia. This suggests a longstanding nutritional impairment in these patients, especially since albumin levels may be normal in the context of prolonged malnutrition (including in the case of marasmus) due to adaptive changes [51,52].

The values of nutrient energy contribution for fat, protein, and carbohydrates fell within the acceptable distribution ranges for all three malnutrition risk categories (MUST), indicating that it was not a prominent aspect in the development of malnutrition [14]. Although in participants with a low malnutrition risk we found an increased energy contribution for carbohydrates, considering the clinical complexity of the disease and the numerous predisposing factors for nutritional decline, it is unlikely that higher carbohydrate intake had a notable “protective” role. Unexpectedly, the high malnutrition risk category had the highest nutrient energy contribution for dietary fats of all three MUST groups (although without a statistical confirmation of the results). This finding raises the question of lipid malabsorption being present in this particular subgroup, and therefore indicates the need for extensive paraclinical investigations of gastrointestinal involvement [53,54,55]. Nevertheless, longitudinal studies could provide a clear perspective on the impact of nutrient energy contributions on weight loss and malnutrition risk in scleroderma.

Gastrointestinal manifestations remain frequent and have been linked to nutritional decline and mortality risk in patients with scleroderma [56,57,58]. Digestive symptoms were overall more frequently found in dcSSc, with dysphagia also being significantly more prevalent in this subgroup. Previously published research describing the prevalence and severity of gastrointestinal involvement according to disease phenotype has reported discrepant results, with some studies identifying more frequent or more severe digestive symptoms in lcSSc [59,60].

Patients with scleroderma obtain diet- and nutrition-related information from various sources, each demonstrating both advantages and disadvantages [61]. Eating habits (including the consumption of late-night meals) may influence the frequency and severity of GERD-associated symptoms [62]. Patients with GERD were significantly more likely to avoid nighttime meals and snacks, a measure that was probably self-imposed given that the current international recommendations for the management of SSc do not include it [63], and none of the patients had undergone professional nutritional counseling. In addition, we found a significant relationship between the presence of GERD and higher intakes of fats and oils, which indicates that the participants’ digestive symptoms were notably impacted by their diet, much like non-scleroderma patients [64]. This aspect can be regarded as an important argument in favor of nutritional interventions that could reduce the burden of gastrointestinal symptoms in scleroderma.

Although the presence of dysphagia was linked to the risk of malnutrition, those patients reported higher caloric intakes compared to rest of the participants, suggesting that energy consumption was not the main contributor to nutritional decline in that subgroup. Moreover, the subjects with dysphagia consumed higher amounts of sugars, preserves, and snacks. Interestingly, calorie-dense, low-consistency foods have been shown to be preferred by persons with dysphagia in previous studies [65].

Decreased appetite was significantly associated with underweight and higher MUST scores. The relationship between the lack of appetite and impaired nutritional status have also been described in other chronic diseases as well as in acute conditions, sometimes regardless of gastrointestinal involvement [66,67,68].

We found that the presence of early satiety was associated with a lower body mass, underweight, unintentional weight loss, and a high risk of malnutrition. Relative to the latter, similar results were obtained by Baron et al. in a study of over 500 patients with scleroderma, which also used MUST as an indicator of malnutrition risk [19,69].

The subjects who were either underweight or eutrophic according to BMI were significantly more likely to exhibit suboptimal energy intakes, thus suggesting that the patients’ diet cannot be excluded as a factor impacting body composition [70]. However, the participants who had experienced weight loss did not demonstrate significantly different mean energy or macronutrient intakes compared to their counterparts. The latter finding could partially explain the absence of a notable relationship between MUST scores and caloric or macronutrient consumption. Moreover, this points to important non-dietary factors being involved in the appearance of weight loss in our study group [2,3,4]. In this respect, we obtained statistically significant connections with mRSS and other disease-related changes.

RFM is calculated differently according to gender and has been shown to predict whole body adiposity more accurately than BMI (which is considered a less reliable index with respect to the assessment of body composition) [15]. Moreover, its variations may have a predictive role regarding the appearance of fatty liver disease, as indicated by the results obtained in a study involving 9967 adults [71]. In our group, RFM was correlated with liver enzyme levels, as well as serum albumin. Additionally, the mean alcohol consumption was also linked to the calculated RFM values. To our knowledge, this is the first study to describe RFM in scleroderma patients.

The intake of nuts and seeds was associated with better liver function and a lower risk of non-alcoholic fatty liver disease in a large cohort of non-Mediterranean American adults [72]. Interestingly, we found a significant inverse correlation between liver transaminase levels and the consumption of nuts and seeds. Although our study did not focus on the benefits of nut consumption on liver function in patients with SSc, this could represent a research perspective (with a prior comprehensive evaluation of the potential subjects’ gastrointestinal health status by a gastroenterologist).

Blood lipid values were correlated with higher mean intakes of fats and oils, as well as central adiposity. Although these are well-known associations in obesity and various other conditions [73,74], few articles focus on the impact of dietary factors on the serum lipid titers in autoimmune diseases, particularly in SSc patients [75,76]. The Mediterranean diet is a food plan that includes a contribution of up to 40% of fats to the total caloric intake [14], yet may reduce body weight, abdominal adiposity, and transaminase levels in patients with metabolic syndrome [77]. Notably, none of the patients in our study group were following the Mediterranean food plan, and only two participants reported using olive oil daily (data not presented). In healthy subjects, it has been shown that adherence to the Mediterranean diet may result in an improvement in microvascular function, raising the question of the potential benefits of dietary interventions in SSc beyond the improvement in lipid levels, blood transaminases, or body composition [78,79].

Vitamin D levels were low in a large proportion of the study group, whereas a small number of patients consumed supplements daily and only two participants had adequate intakes of dietary vitamin D. In a retrospective cohort of scleroderma patients, Trombetta et al. evaluated the effects of supplementation on serum vitamin D concentrations, obtaining no significant correlations with the latter [80]. Similar to previously reported findings, vitamin D was correlated with the extent of skin involvement in our group [81]. However, results remain discrepant across studies in this respect [82].

Beyond the consequences of an unbalanced diet, unintentional weight loss and malnutrition risk have been linked to a plethora of potential risk factors, including cardiopulmonary and gastrointestinal involvement, systemic inflammation, mood disturbances or depression, and functional impairment [18,19,83]. Other issues such as microstomia and tooth loss may also influence scleroderma patients’ nutritional status [84,85]. Importantly, malnutrition was found to increase the risk of mortality in the general population as well as in patients with scleroderma. These results raise the issue of providing effective strategies for the prevention of weight loss and malnutrition in SSc [8,86,87]. Through intricate mechanisms, chronic systemic diseases are often associated with nutritional status alterations [88,89,90,91]. We found that most patients demonstrated a lower body mass on evaluation for the present study compared to the one recorded at diagnosis. Moreover, BMI values over 25 kg/m^2^ were significantly less prevalent at the time of examination than those recorded at the moment of diagnosis. Additionally, underweight (BMI < 18.5 kg/m^2^) was not present at the moment of SSc diagnosis in our study group, suggesting that scleroderma patients are at risk of nutritional decline secondary to disease-related manifestations [2,21].

Notably, advanced age did not play a decisive role in increasing malnutrition risk in our study group. Although it has been shown that malnutrition is more prevalent in elderly persons due to both socioeconomic and health-related factors [92,93,94], previously published research involving scleroderma patients also describes a lack of statistical significance between age and nutritional decline [18,19].

## 5. Conclusions

Patients with scleroderma have been shown to be at risk of nutritional decline, yet the scientific evidence pertaining to the predisposing factors and the management of unintentional weight loss and malnutrition remains scarce. Together with disease-related changes such as digestive involvement and cardiopulmonary manifestations, an unbalanced diet may also contribute to the appearance of weight loss and malnutrition. We found intricate relationships between dietary factors, eating habits, unintentional weight loss, malnutrition risk, and digestive symptoms, as well as cardiopulmonary involvement in our study group. Currently, there is an unmet need for longitudinal and interventional research focusing on the long-term significance, ramifications, and management of nutritional impairment in SSc patients with various clinical manifestations. Our results indicate that scleroderma patients could benefit from personalized nutritional counseling in an interdisciplinary setting.

## Figures and Tables

**Table 1:** Characteristics of the study group.

Parameter (*n* = 42)	Mean (±SD)/Number (%)			
General Characteristics				
Age (years)	51.67 (±12.42)			
Gender	Female 36 (85.7%)		Male 6 (14.3%)	
Area of residence	Urban 24 (57.1%)		Rural 18 (42.1%)	
Disease phenotype	dcSSc 18 (42.9%)		lcSSc 24 (57.1%)	
mRSS	12.45 (±9.26)			
Duration	0–5 years 15 (35.7%)	6–10 years 11 (36.2%)		>10 years 16 (38.1%)
EUSTAR-AI	2.22 (±1.73)			
Autoantibodies				
Anti-topoisomerase I	17 (40.5%)			
Anti-centromere	24 (57.1%)			
Anti-RNA polymerase III	1 (2.4%)			
ANA	38 (90.5%)			
Organ Involvement				
PAH	9 (21.4%)			
ILD	22 (52.4%)			
CHF	7 (16.7%)			
CKD	6 (14.29%)			
Arrythmias and conduction defects	11 (26.2%)			
Gastrointestinal symptoms	34 (81%)			
Anthropometric Parameters				
H (cm)	164.95 (±7.45)			
W (kg)	64.44 (±13.09)			
W_dg_ (kg)	76.09 (±14.14)			
AC (cm)	82.31 (±13.21)			
HC (cm)	101.58 (±8.86)			
WHR	0.80 (±0.09)			
BMI (kg/m^2^)	23.63 (±4.14)			
BMI_dg_ (kg/m^2^)	27.94 (±4.72)			
RFM	33.42 (±6.72)			
MAC (cm)	31.71 (±5.11)			

AC: abdominal circumference; ANA: antinuclear antibodies; BMI: body mass index; BMI_dg_: BMI noted at the moment of diagnosis; CHF: chronic heart failure; CKD: chronic kidney disease; EUSTAR-AI: European Scleroderma Trials and Research group Activity Index; H: height; HC: hip circumference; ILD: interstitial lung disease; MAC: mid-arm circumference; mRSS: modified Rodnan skin score; PAH: pulmonary arterial hypertension; RFM: relative fat mass; RNA: ribonucleic acid; W: weight; W_dg_: body mass recorded at diagnosis; WHR: waist/hip ratio.

**Table 2:** Biochemical parameters.

Parameter (*n* = 42)	Min	Max	Mean	SD
Albumin (mg/dL)	32.30	54.70	39.26	4.13
Total protein (g/dL)	6.01	9.41	7.25	0.62
Vitamin D (ng/mL)	2.52	33.89	10.39	10.42
CRP (mg/dL)	0.03	5.10	1.16	1.23
AST (mg/dL)	10.00	36.50	20.55	5.99
ALT (mg/dL)	6.43	49.00	19.33	9.57
Creatinine (mg/dL)	0.49	1.31	0.78	0.21
Total cholesterol (mg/dL)	98.00	280.00	180.55	47.87
Triglycerides (mg/dL)	37.80	202.60	100.42	44.12

ALT: alanine transaminase; AST: aspartate aminotransferase; CRP: C-reactive protein.

**Table 3:** The average daily consumption of various foods and beverages in patients with and without weight loss.

Foods and Beverages (g/day) (Mean ± SD)	Total	Unintentional Weight Loss		*p*
		Present	Absent	
Cereals and cereal products	217.39 ± 99.59	214.93 ± 96.81	219.06 ± 103.39	0.896
Fruits	412.97 ± 360.05	296.84 ± 215.30	491.94 ± 417.75	0.085
Vegetables	213.66 ± 164.85	172.10 ± 96.86	241.92 ± 195.24	0.134
Meat and meat products	141.12 ± 151.22	197.34 ± 218.80	102.89 ± 58.21	0.100
Fish and fish products	27.08 ± 22.84	30.32 ± 26.10	24.88 ± 20.60	0.477
Dairy	390.04 ± 212.26	366.60 ± 194.11	405.99 ± 226.27	0.550
Eggs and egg dishes	18.02 ± 13.07	16.15 ± 10.51	19.30 ± 14.63	0.421
Fats and oils	17.55 ± 13.39	19.30 ± 12.89	16.36 ± 13.86	0.487
Sweets, preserves, snacks	31.48 ± 22.56	25.85 ± 29.60	39.77 ± 14.26	0.048
Nuts and seeds	6.74 ± 9.06	3.94 ± 6.09	8.64 ± 10.31	0.099
Potatoes	67.95 ± 55.25	84.65 ± 57.01	56.61 ± 52.12	0.115
Soups	256.44 ± 204.29	348.48 ± 250.34	193.86 ± 139.42	0.014
Non-alcoholic beverages other than water	482.83 ± 331.92	464.70 ± 348.53	495.16 ± 326.86	0.777
Alcoholic beverages	11.86 ± 33.36	17.01 ± 47.18	8.35 ± 19.63	0.483

**Table 4:** The average daily consumption of energy and macronutrients in the study group (total and with respect to the presence of weight loss).

Energy and Macronutrients (Mean ± SD)	Total	Unintentional Weight Loss		*p*
		Present	Absent	
Energy (kcal)	1944.05 ± 844.82	2153.52 ± 822.56	1801.61 ± 571.81	0.246
Protein (g)	90.64 ± 50.93	104.47 ± 73.81	81.23 ± 23.89	0.226
Protein (kcal)	355.44 ± 204.97	417.88 ± 295.25	312.99 ± 94.95	0.174
Protein (% of energy intake)	18.67 ± 3.46	19.22 ± 3.74	18.29 ± 3.28	0.412
Lipids—total (g)	72.84 ± 37.19	83.78 ± 48.89	65.40 ± 25.03	0.117
Lipids (kcal)	655.53 ± 334.70	754.01 ± 440.02	588.56 ± 225.29	0.167
Lipids (% of energy intake)	33.31 ± 6.13	34.49 ± 5.56	32.51 ± 6.47	0.295
MUFA (g)	25.89 ± 13.73	29.92 ± 18.08	23.15 ± 9.21	0.118
PUFA (g)	13.45 ± 7.30	15.55 ± 9.64	12.03 ± 4.90	0.127
SFA (g)	27.20 ± 14.80	31.92 ± 18.79	23.98 ± 10.60	0.088
Cholesterol (g)	384.73 ± 187.17	447.77 ± 256.51	341.86 ± 106.08	0.071
Carbohydrates—total (g)	241.94 ± 100.17	252.74 ± 116.28	234.59 ± 89.38	0.591
Carbohydrates (kcal)	967.75 ± 400.68	1010.95 ± 465.12	938.38 ± 357.51	0.571
Carbohydrates (% of energy intake)	50.26 ± 8.60	47.87 ± 8.29	51.89 ± 8.58	0.137
Fructose (g)	31.13 ± 21.41	29.18 ± 20.95	32.46 ± 22.04	0.629
Galactose (g)	0.96 ± 0.87	0.87 ± 0.88	1.03 ± 0.87	0.575
Glucose (g)	25.07 ± 16.99	27.44 ± 20.38	23.46 ± 14.49	0.492
Lactose (g)	17.67 ± 9.48	16.37 ± 8.49	18.55 ± 10.17	0.456
Maltose (g)	2.13 ± 1.62	2.68 ± 2.25	1.76 ± 0.88	0.073
Starch (g)	102.56 ± 41.29	113.31 ± 46.48	95.25 ± 36.53	0.189
Sucrose (g)	58.51 ± 27.90	61.18 ± 32.02	56.70 ± 25.27	0.633
Total sugars (g)	138.54 ± 68.91	141.47 ± 77.52	136.55 ± 64.01	0.830

MUFA: monounsaturated fatty acids; PUFA: polyunsaturated fatty acids; SFA: saturated fatty acids.

**Table 5:** The average daily consumption of micronutrients in the study group (total and with respect to the presence of weight loss).

Micronutrient (Mean ± SD)	Total	Unintentional Weight Loss		*p*
		Present	Absent	
Calcium (mg)	899.01 ± 368.57	894.01 ± 369.56	902.41 ± 375.47	0.943
Chloride (mg)	4726.31 ± 2307.33	5602.86 ± 2890.13	4130.24 ± 1616.13	0.041
Copper (mg)	1.58 ± 0.94	1.82 ± 1.29	1.41 ± 0.55	0.161
Iodine (mg)	153.65 ± 60.23	161.41 ± 66.95	148.35 ± 56.01	0.513
Iron (mg)	10.87 ± 4.91	12.01 ± 6.64	10.08 ± 3.19	0.271
Magnesium (mg)	291.05 ± 116.60	293.79 ± 143.49	289.18 ± 97.41	0.909
Manganese (mg)	2.90 ± 1.23	2.58 ± 1.22	3.11 ± 1.21	0.178
Nitrogen (mcg)	14.57 ± 8.13	16.67 ± 11.81	13.14 ± 3.82	0.248
Phosphorus (mg)	1410.47 ± 612.38	1512.21 ± 824.09	1341.27 ± 419.01	0.439
Potassium (mg)	3576.92 ± 1636.08	3686.49 ± 1996.31	3502.40 ±1379.01	0.744
Selenium (mcg)	74.71 ± 33.55	80.95 ± 46.44	70.47 ± 20.96	0.393
Sodium (mg)	3143.18 ±1563.81	3754.11 ± 1963.09	2727.73 ± 1079.08	0.035
Zinc (mg)	9.90 ± 5.96	11.74 ± 8.57	8.65 ± 2.75	0.168
Total folate (mcg)	262.67 ± 120.92	255.14 ± 116.11	267.77 ± 126.18	0.740
Carotene (total) (mcg)	3456.39 ± 2294.82	2729.51 ± 1495.25	3950.67 ± 2622.43	0.063
Alpha-carotene (mcg)	489.52 ± 381.31	370.98 ± 368.97	570.12 ± 375.37	0.097
Beta-carotene (mcg)	2991.77 ± 2030.92	2336.63 ± 1257.51	3437.25 ± 2341.21	0.056
Vitamin A (retinol) (mcg)	1413.84 ± 1450.35	1801.10 ± 1872.36	1150.50 ± 1038.19	0.156
Vitamin A—retinol equivalents (mcg)	1996.54 ± 1472.35	2270.95 ± 1925.53	1809.94 ± 1068.63	0.379
Vitamin B1(thiamin) (mg)	1.54 ± 0.91	1.71 ± 1.31	1.42 ± 0.49	0.402
Vitamin B2 (riboflavin) (mg)	1.97 ± 0.87	2.06 ± 1.14	1.89 ± 0.63	0.601
Vitamin B3 (niacin) (mg)	25.45 ± 14.36	30.33 ± 20.65	22.13 ± 6.30	0.069
Vitamin B6 (pyridoxine) (mg)	2.08 ± 1.11	2.25 ± 1.49	1.97 ± 0.75	0.488
Vitamin B12 (cobalamin) (mcg)	8.16 ± 6.30	10.08 ± 8.61	6.84 ± 3.73	0.102
Vitamin C (ascorbic acid) (mg)	120.69 ± 94.70	107.28 ± 80.01	129.81 ± 104.12	0.433
Vitamin D (ergocalciferol) (mcg)	2.52 ± 1.56	2.91 ± 2.05	2.26 ± 1.07	0.245
Vitamin E(α-tocopherol equivalents) (mg)	12.40 ± 6.21	13.07 ± 7.17	11.94 ± 5.57	0.591

## Data Availability

Not applicable.
